# Enhanced Gene Silencing through Human Serum Albumin-Mediated Delivery of Polyethylenimine-siRNA Polyplexes

**DOI:** 10.1371/journal.pone.0122581

**Published:** 2015-04-09

**Authors:** Elena Nicolì, Marie Isabel Syga, Michela Bosetti, V. Prasad Shastri

**Affiliations:** 1 Dipartimento di Scienze del Farmaco, University of Eastern Piedmont, Novara, Italy; 2 Institute for Macromolecular Chemistry, University of Freiburg, Freiburg, Germany; 3 BIOSS–Centre for Biological Signalling Studies, University of Freiburg, Freiburg, Germany; National University of Ireland, Galway, IRELAND

## Abstract

Small interfering RNA (siRNA) targeted therapeutics (STT) offers a compelling alternative to tradition medications for treatment of genetic diseases by providing a means to silence the expression of specific aberrant proteins, through interference at the expression level. The perceived advantage of siRNA therapy is its ability to target, through synthetic antisense oligonucleotides, any part of the genome. Although STT provides a high level of specificity, it is also hindered by poor intracellular uptake, limited blood stability, high degradability and non-specific immune stimulation. Since serum proteins has been considered as useful vehicles for targeting tumors, in this study we investigated the effect of incorporation of human serum albumin (HSA) in branched polyethylenimine (bPEI)-siRNA polyplexes in their internalization in epithelial and endothelial cells. We observed that introduction of HSA preserves the capacity of bPEI to complex with siRNA and protect it against extracellular endonucleases, while affording significantly improved internalization and silencing efficiency, compared to bPEI-siRNA polyplexes in endothelial and metastatic breast cancer epithelial cells. Furthermore, the uptake of the HSA-bPEI-siRNA ternary polyplexes occurred primarily through a caveolae-mediated endocytosis, thus providing evidence for a clear role for HSA in polyplex internalization. These results provide further impetus to explore the role of serum proteins in delivery of siRNA.

## Introduction

Gene therapy is a therapeutic approach that aims to treat otherwise incurable diseases, like viral infections, hereditary disorders and cancer by replacing defective genes and the function of aberrant proteins, through gene incorporation in plasmids or viral vectors for nuclear delivery [1]. Non-viral systems are however widely preferred, in order to reduce cellular toxicity, risk of casual gene insertion in the genome and mutagenesis. More recently, the discovery of endogenous mechanisms involved in regulating gene expression, through antisense oligonucleotides has opened a new approach for gene therapy, i.e., targeting a specific gene for silencing in cells. RNA interference (RNAi) is a mechanism triggered in the cell cytoplasm by exogenous small interfering RNAs (siRNAs) and endogenous microRNAs (miRNAs), causing the blockade of protein translation, by specific mRNA sequence matching [1–2]. During embryogenesis and development, miRNAs, which are “nearly” matched sequences coded by the cell genome, are involved in the regulation of the expression of multiple genes. miRNA expression is tightly regulated in different tissues and the sequences are conserved across species, indicating that they have a fundamental role in supervising cell functions [3]. The endogenous role of miRNAs makes their application in gene therapy rather challenging, since multiple genes can be regulated by a single miRNA and multiple miRNAs can regulate the expression of a specific gene. siRNAs, on the contrary are perfectly matched sequences that can be designed to target a specific mRNA. Their activity in cells was identified as part of an antiviral response, where retroviral RNA is recognized in the cell cytoplasm by the RNAi mechanism [4]. Particularly important is the activation of the RNAi silencing complex (RISC) that recognizes and binds double stranded siRNAs and miRNAs, through a similar mechanism. Argonaute proteins, which reside within the RISC complex, unwind siRNAs and miRNAs, allowing RISC to selectively bind the antisense strand, using that as a template to recognize the target mRNAs in the cytoplasm. The imperfect match of miRNAs leads mostly to translational repression, while siRNA’s perfect match causes mRNA cleavage and degradation [2–3]. The design of synthetic siRNA sequences of 21–25 bp, is nowadays employed for specific gene knockdown, with increasing number of RNAi-based drugs in clinical trials [5–6].

The delivery of siRNA presents many challenges specifically with regards to *in vivo* injection and intracellular uptake as siRNA sequences are negatively charged and do not spontaneously cross the cell membrane. Furthermore, major issues to be considered include degradation by extracellular nucleases, low blood circulation, renal clearance and difficulties in traversing the endothelial barrier to reach a target tissue [7–2].

The condensation of siRNA with linear or branched cationic polymers is widely employed as a packaging and delivery strategy, as this ensures protection of the siRNA from degradation while promoting non-specific endocytosis, and intracellular escape from endosomes [7–8]. Polycations, although commonly used for condensation and in vivo delivery of siRNA, have associated toxicity, with an inverse relationship between backbone charge density and toxicity [9]. The first cationic polymer-siRNA formulation, to enter clinical trial in 2008, was based on cyclodextrin based nanoparticles containing siRNA (CALAA-01) with incorporation of transferrin (Tf), as a targeting ligand for tumors [6]. Transferrin transports iron in the bloodstream and malignant cells have an up-regulated expression of the Tf-receptor [10].

Serum proteins have recently been discovered to serve as endogenous targeting ligands and to play a role in increasing nanoparticle circulation [5–11]. Recently, Kim *et al*., have reported that the adsorption of apolipoprotein A-I, a component of the high density lipoprotein (HDL) by liposomes during circulation enables liver targeting of siRNA through a specific receptor-mediated internalization process in hepatocytes [12]. Therefore, identifying other possible endogenous targeting molecules in circulation can provide new insights and opportunities for siRNA delivery.

Albumin, which is the most abundant protein with physiological function of transporting fatty acids, has been explored extensively for the delivery of therapeutic molecules. Most notable is the enhanced delivery and efficacy of paclitaxel when delivered as an albumin conjugated nanoparticle, Abraxane [11]. Additionally, it has been shown that albumin can accumulate in tumors through the enhanced permeability and retention mechanism and via specific trans-endothelial transport processes, and is therefore promising as a carrier for anti-cancer drugs [13–14]. Work to date, on using albumin as a carrier for gene delivery, has focused on the covalent modification of the albumin molecule to synthesize cationized albumin (CA) [15] or on the complexation of human serum albumin (HSA) with cationic polymers, to enhance DNA complexation and cell transfection. Poly-L-lysine-coated albumin nanoparticles for example, were shown to increase siRNA complexation and resistance to enzymatic degradation [16]. Furthermore, some earlier studies have reported the use of polyethylenimine (PEI)-HSA-DNA nanocomplexes for gene delivery in cells [17–18]. Abbasi et al., have recently reported the delivery of siRNA by complexation with polyethylenimine (PEI)-coated human serum albumin (HSA) nanoparticles [19]. Son et al., have introduced the concept of using HSA to guide siRNA delivery to tumors, through nanocarriers formed by disulfide-crosslinking between thiolated HSA and polymerized siRNA, and have reported increased in vivo accumulation of the nanocarriers at the tumor sites [20].

Encouraged by these findings, in this study, we explored the role of native unmodified HSA in mediating the delivery of siRNA in endothelial and breast cancer metastatic cells. Branched polyethylenimine (bPEI), the most commonly used cationic polymer for nucleic acids delivery, was complexed with siRNA and HSA was added subsequently for electrostatic incorporation via bPEI [21–22]. HSA-bPEI-siRNA ternary complexes significantly improved internalization and silencing efficiency, compared to bPEI-siRNA polyplexes. The presence of free albumin interfered with both internalization and silencing, suggesting an important role for HSA in mediating transfection. The participation of HSA in intracellular trafficking was further elucidated by uptake mechanism studies.

## Materials and Methods

### Materials

TurboGFP *Stealth* RNAi *siRNA sequence was* designed to target TurboGFP mRNA, with the on-line program BLOCK-iT RNAi Designer (http://rnaidesigner.lifetechnologies.com/). TurboGFP *Stealth* RNAi *siRNA* was used for the characterization studies of the complexes and the silencing experiments. Sense sequence (5’-3’): *GAUAACGAUCUGGAUGGCAGCUUCA*, antisense sequence (5’-3’): *UGAAGCUGCCAUCCAGAUCGUUAUC*.

BLOCK-iT AlexaFluor 555 control siRNA (Invitrogen, Germany) was used for all the uptake and mechanism studies. Branched polyethylenimine (MW 25 kDa) and Human serum albumin were purchased from Sigma-Aldrich (Germany). Lipofectamine2000 was purchased from Invitrogen (Germany).

### Cell culture

Human primary pulmonary microvascular endothelial cells (HPMEC) and human breast adenocarcinoma cell line (MDA-MB-231) were used for all the siRNA transfection studies. MDA-MB-231 cells were provided by the Centre for Biological Signalling Studies (BIOSS) and were genotyped and verified at Labor für DNA Analytik (Freiburg, Germany), while HPMEC were purchased from ScienCell (Provitro, Germany).

MDA-MB-231 were grown in Dulbecco’s modified Eagle’s Medium (DMEM) (Invitrogen, Germany) supplemented with 10% FBS (Invitrogen, Germany) and 1% penicillin/streptomycin/amphotericin B solution (Pan Biotech, Germany) and HPMEC were cultured in Endothelial Cell Medium (ECM), supplemented with 5% fetal bovine serum (FBS), 1% endothelial cell growth supplement (ECGS) and 1% penicillin/streptomycin/amphotericin B solution (ScienCell, US/Provitro, Germany). Cells were cultured in a humidified incubator containing 5% CO_2_ at 37°C.

TurboGFP expressing MDA-MB-231 and HPMEC were obtained by stable transfection of TurboGFP using lentiviral transfection and the non-silencing control was obtained using the pGIPZ vector (Thermo Scientific, Germany). MDA-MB-231 and HPMEC were seeded in 6 well plates at a concentration of 7.0*10^4^cells/well and transfected with the lentiviral solution in complete medium for 24 hours. Puromycin (Sigma-Aldrich, Germany) was added at the concentration of 4μg/ml after 48 hours, to select the successfully transfected population.

### Synthesis of polyplexes

The ternary complexes were prepared in DNAse-RNAse free water (GIBCO, Germany). SiRNA was mixed with branched polyethylenimine (bPEI, 25kDa) at nitrogen/phosphate group ratio (N/P ratio) of 10 and incubated at room temperature for 20 minutes. bPEI solution and human serum albumin (HSA, 67 KDa) solution were prepared in DNAse-RNAse free water and filtered with a 0.22 μm cellulose acetate filter (Corning,NY) immediately prior to use. The complexation reaction (step) was performed with a fixed siRNA concentration of 22 μg/ml BLOCK-iT Alexa Fluor 555 control siRNA (~13.8 kDa) or 26 μg/ml of *Stealth* RNAi TurboGFP *siRNA* sequence (~16.5 kDa). HSA was added at a final concentration of 0,125 mg/ml and the system was allowed to react for a further 30 minutes. Lipofectamine2000 (Invitrogen, Germany) was used as a positive control, for the uptake and silencing experiments. The lipoplexes were prepared with the same siRNA concentration used for HSA-bPEI-siRNA ternary complexes and bPEI-siRNA polyplexes, following the protocol guide from Invitrogen, in relation to the surface area and in relation to cell density (2.8 μl).

### Gel Retardation assay and RNAse protection assay

Gel retardation assay and RNAse protection assay were performed loading 10 μl aliquot of the sample, together with 2μl of loading buffer (Invitrogen, Germany) on a 4% agarose gel, prepared in 1X Tris-boric acid-EDTA (TBE) buffer. Protection against RNase was assessed by incubating the samples for 30 minutes with 17μg/ml of RNAse solution (Sigma-Aldrich, Germany) followed by gel electrophoresis. The Electrophoresis was carried out in TBE buffer at 100V for 1 hour. Gel bands were stained with Gel red nucleic acid stain (Biotium, US) for 30 minutes and visualized under UV Fusion FX7 (PeqLab). A 100bp and 10bp DNA ladder (Invitrogen, Germany) was included in every run to confirm siRNA integrity.

### Determination of Size and Zeta Potential

The mean size of the ternary complex, polydispersity, zeta potential, and stability, were measured by dynamic light scattering (DLS), using a DelsaNano C particle analyzer (Beckman Coulter). The complexes were diluted 1:3 with distilled DNAse-RNAse free water for measurement. The stability of the ternary complexes was determined by size measurement at the time-point of 30 minutes, 1 hour, 2 hours and 4 hours. Morphology and size of the complexes were further characterized by transmission electron microscopy (TEM) (Zeiss LEO 912 Omega TEM) at an accelerating voltage of 120 kV. The prepared samples was settled on a CF-400-Cu square mesh copper grid (Electron Miscroscopy Sciences, USA) and stained with 2% uranil acetate solution. ImageJ software was used to create a statistic of the size of the complexes.

### Microscopy of transfection studies

Cell microscopy studies were performed in 8-well chambers (Sarstedt, Germany). 1.5*10^4^ cells were seeded 24 hours before the experiment in 300 μl of complete medium. Alexa Fluor 555 siRNA-labeled complexes were incubated at 37°C for 4 hours. The cells were washed 3 times with Phosphate Buffered Saline *PBS* (GIBCO, Germany) and then were fixed in 4% formaldehyde solution and stained with DAPI using Vectashield Mounting Medium (Vector Laboratories, Burlingame). Images were acquired in multichannel acquisition mode with Axio Observer Z1 (Zeiss), with a 20 X objective and the images were processed using Axio vision software.

### Quantification of transfection studies using fluorescence activated cell sorting (FACS)

Quantitative siRNA internalization in cells was studied measuring the mean fluorescence value for cell, following the uptake of siRNA-labeled complexes in MDA-MB-231 and HPMEC. Cells were seeded in 24-well plate at a concentration of 1.0*10^5^ in 500 μl of complete medium, 24 hours before the experiment. Cells were then transfected with the complexes for 4 hours in 500μl of serum free medium. Subsequently, cells were washed with PBS and detached with trypsin from each well. Complete medium with 10% FBS was used to block trypsin action. The cells were centrifuged at 800 rpm 5 minutes and the pellet was resuspended in PBS 2% FBS. The fluorescent signal was detected with FACS Gallios (Beckman Coulter) with FL2 channel (excitation laser: 488 nm; emission filter: 575 nm) and the data were analyzed with Flowing software 2 (Perttu Terho). Histograms were generated using FlowJo software.

### Silencing experiments

Silencing experiments were conducted with MDA-MB-231 and HPMEC expressing TurboGFP. Cells were seeded in 24-well plate at a concentration of 2.0*10^4^ in in 500 μl of complete medium, 24 hours before the experiment. Cells were transfected with the complexes for 4 hours in 500μl of serum free medium. The medium was replaced with complete medium, containing FBS, for 72 hours. Cells were then washed with PBS and detached with trypsin from each well. Complete medium with 10% FBS was used to block trypsin action. The cells were centrifuged at 800 rpm 5 minutes and the pellet was resuspended in PBS 2% FBS. The fluorescent signal was detected with FACS Gallios (Beckman Coulter) with FL1 channel (excitation laser: 488 nm; emission filter 525 nm) and the data were analyzed with Flowing software 2 (Perttu Terho). Lypofectamine2000 (Invitrogen) was used as a positive control. Histograms were generated using FlowJo software.

### Cell Viability Assay

To test the cytotoxicity of the different formulations, an 3-(4,5-Dimethylthiazol-2yl)2,5-diphenyl-2H-tetrazoliumbromide (MTT) assay (Sigma, Germany) was executed after the incubation with the complexes. Cells were seeded in a 24-well plate at a concentration of 2.0*10^4^ in in 500 μl of complete medium, 24 hours prior to the experiment. The cells were transfected with the complexes for 4 hours in serum free medium. Then the serum free medium was replaced with complete medium for 72 hours, testing the cell viability at the same time point of the knockdown efficiency. Cells were then washed with PBS and 200 μl of a solution of MTT (0.25 mg/ml in RPMI without phenol red) was added and incubated for 3 h at 37°C. The MTT solution was removed, 200 μl of DMSO were added to each well and the absorbance at 550 nm was measured using a Synergy HT plate reader (BioTek).

### Uptake mechanism studies

Colocalization studies with selective markers of endocytosis were conducted in both cell lines. Alexa-Fluor 647 labeled transferrin (Tf) (50μg/ml) and Cholera toxin b (CTB) (3.5μg/ml) (Invitrogen, Germany) were used as intracellular markers of clathrin-mediated endocytosis and caveolae-mediated endocytosis. Cells were transfected with Alexa-Fluor 555 siRNA-labeled complexes for 2 hours and transferrin (Tf) or cholera toxin b (CTB) were subsequently added for 30 minutes. Inhibition studies were performed with specific inhibitors: Chlorpromazine (2μg/ml for MDA-MB-231 and 5μg/ml for HPMEC), Filipin III (5μg/ml) and Nystatin (20μg/ml). All the inhibitors were purchased from Sigma-Aldrich. Cells were pre-treated for 20 minutes with each inhibitor, in serum free medium, and then incubated with the siRNA-labeled complexes for 2 hours, without removing the inhibitors. Cells were then trypsinized and collected for FACS fluorescence detection, using FL2 channel (excitation laser: 488 nm; emission filter: 575 nm), and the data were analyzed with Flowing software 2 (Perttu Terho). The chosen concentrations of each inhibitor were previously tested for cell viability with MTT test (S3).

### Statistical analysis

Statistics were carried using the student t-test module in Excel for paired datasets, assuming a two-tailed distribution and equal variance between data sets (homoscedastic). A p value of < 0.05 was considered statistically significant.

## Results and Discussion

### Characterization of HSA-bPEI-siRNA ternary complex

**Fig 1:**
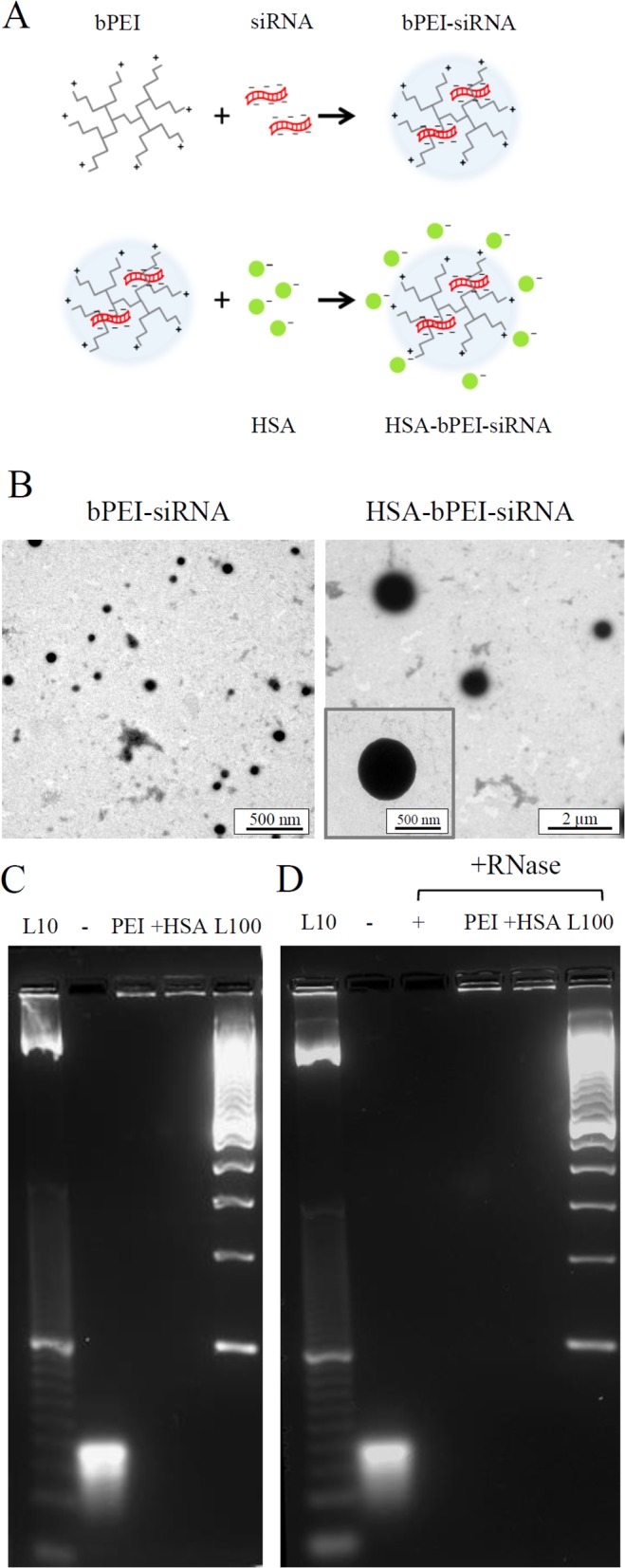
Characterization of HSA-bPEI-siRNA complexes. A) Schematic representation of the two steps formulation protocol, where bPEI-siRNA native interaction was preserved and HSA was used for interacting with the positive charged amino-groups of bPEI. B) Transmission electron microscopy (TEM) images showing the increase in size upon addition of HSA. C) Gel retardation assay, demonstrating siRNA complexation on the top of the gel. From left, 10 base pair ladder (L10); free siRNA (-); PEI-siRNA (PEI); HSA-PEI-siRNA (+HSA); 100 base pair ladder (L100). D) RNAse protection assay, indicating efficent siRNA protection from RNAse degradation. Complexes are unaltered on the top of the gel, while free RNA is completely degraded. From left, 10 base pair ladder (L10); free siRNA (-); samples incubated with RNAse: free siRNA (+), PEI-siRNA (PEI) and HSA-PEI-siRNA (+HSA); 100 base pair ladder (L100).

Due to the negatively charged backbone, delivery of siRNA to cells requires it to be packaged as a complex with a polycation. Typically, PEI (linear or branched) is the polycation of choice. This complexation is important to ensure the stability of the siRNA through RNAses and to promote the entry of siRNA into cells. The polyplexes were formed using branched polyethylenimine (bPEI) and HSA was introduced as an aqueous solution at pH 7, where it is negatively charged, thereby capable of interacting with the positive charged amino groups of bPEI (Fig 1A). To arrive at the chosen ratio between HSA, bPEI and siRNA, we took into consideration a recent study showed that the ability of bPEI-siRNA at N/P ratio 20 to mediate efficient gene silencing was higher in comparison to N/P ratio of 5 and 10 [23]. Therefore, with the aim to increase bPEI-siRNA activity at lower N/P ratio, we employed for this study bPEI at N/P ratio of 10 as this resulted in a good condensation of the siRNA and internalization by cells. The concentration of HSA that yielded the most measurable change in the size of the polyplexes and an increase in the cell uptake efficiency was identified using serial dilution as the optimal concentration for the ternary polyplex formation. In general the introduction of HSA to bPEI-siRNA polyplexes resulted in a dramatic increase in the size, which was also confirmed by TEM (Fig 1B), and in the reversal of the overall charge of the complexes to a negative value as determined by DLS measurements (Table 1). This increase in size could be due to either aggregation of smaller polyplexes or reorganization of the bPEI-siRNA complexes, in presence of albumin, to a more thermodynamically stable structure. Since the association with albumin resulted in complexes ranging in size from 800–1200 nm with a room temperature stability of about 4 hours (S1 Fig), all the studies were performed with freshly prepared complexes. We then ascertained if the addition of albumin would impact the capacity of siRNA to complex with bPEI. Gel Retardation assay showed complete siRNA incorporation by the complexes (Fig 1C) suggesting that HSA adsorption on nanocomplexes surface did not alter the ability of siRNA to condense. Polyplex formation is known to confer stability against proteolytic degradation [23], and the ability of HSA-bPEI-siRNA to resist degradation by extracellular endonucleases was verified by RNAse Protection assay (Fig 1D).

**Table 1:** Size—zeta potential characterization and polydispersity index value.

	Size (nm) TEM	Size (nm) DLS	PDI	ζ-potential (mV)
**PEI-siRNA**	84 ± 17	103 ± 12	0.2	+ 2.4 ± 4
**HSA-PEI-siRNA**	842 ± 230	959 ± 170	0.2	- 4.5 ± 5

Reported values are an average (n = 12)

### HSA colocalizes with siRNA in cells and increases transfection efficiency

To investigate the cell uptake efficiency of the developed system and to understand the role of HSA in mediating siRNA delivery, we performed the transfection studies in metastatic human breast cancer cells (MDA-MB-231) and *human pulmonary microvascular endothelial cells* (*HPMEC*). These cells were chosen as vasculature is important for tumor survival and lung is the prominent organ for epithelial tumor metastasis [24–25]. Additionally, endothelial cells and metastatic breast cancer cells have been shown to actively take up albumin [26–27].

**Fig 2:**
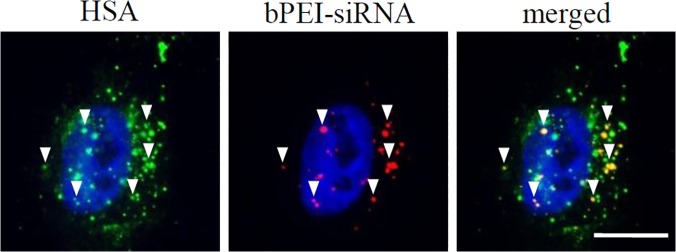
Colocalization of HSA and bPEI-siRNA polyplexes in MDA-MB-231. Fluorescence microscopy revealed that Alexa-fluor 488 labeled HSA and Alexa-fluor 555 siRNA access in the same intracellular trafficking. (DAPI nuclear stain, blue; Alexa-fluor 555 siRNA labeled complexes, red; Alexa-fluor 488 labeled HSA, green). White arrows indicate points of colocalization. Scale bar: 20μm.

To ascertain the role of HSA in the internalization of siRNA, a colocalization study was undertaken, in MDA-MB-231, with fluorescently labeled siRNA. Fluorescence microscopy revealed that HSA colocalizes with siRNA, therefore, clearly shown that HSA enters the cell intact as an ensemble and participates in the transcellular uptake of siRNA complexes and their trafficking in the cytosol (Fig 2).

**Fig 3:**
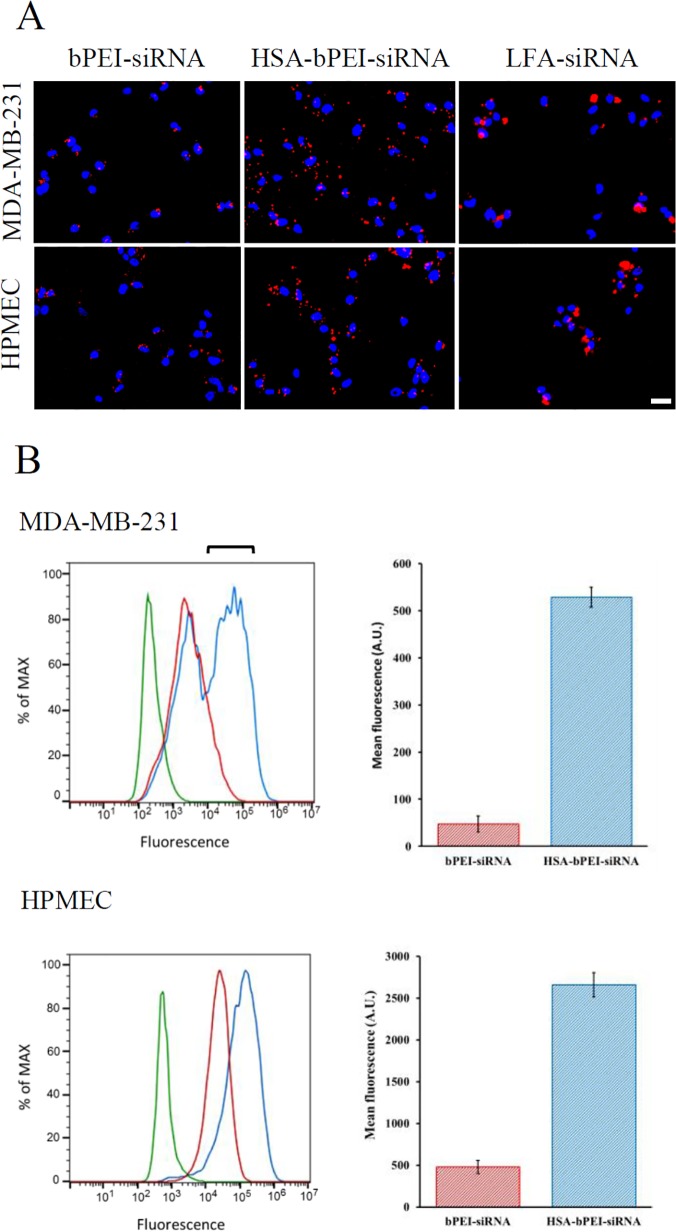
Uptake of HSA-bPEI-siRNA and PEI-siRNA complexes in MDA-MB-231 and HPMEC. A) Representative fluorescence micrographs showing siRNA labeled complexes in cells (DAPI nuclear stain, blue; Alexa-fluor 555 siRNA labeled complexes, red) Scale bar: 20μm. B) FACS quantitative measurements, expressed as mean fluorescence value for cell,reveald increased uptake by HSA-bPEI-siRNA complexes, compared to bPEI-siRNA control, in both cell lines. Results are shown as mean ± SD (n = 3). MDA-MB-231 p<0.001, HPMEC p<0.005.

The quantification of HSA-bPEI-siRNA complex internalization was first evaluated in MDA-MB-231 and HPMEC and compared with bPEI-siRNA polyplexes, using flow cytometry (FACS). Alexa dye labeled siRNA formulations were used for the study. To eliminate the interference from the transfection medium, uptake studies were conducted in serum free medium. Fluorescence micrographs of transfected cells are shown (Fig 3A). FACS analysis showed that the ternary complexes were taken up to a significantly greater extent in comparison to bPEI-siRNA polyplexes. The uptake efficiency was about 11 fold greater, in MDA-MB-231, and about 6 fold greater, in HPMEC, over bPEI-siRNA control. In contrast to HPMEC, where a single gaussian distribution was observed, in MDA-MB-231 cancer cells a bimodal distribution was observed, with about 55% of the population showing higher internalization and the remaining population having similar uptake efficiency of bPEI-siRNA (Fig 3B). It has been known that size, shape and presence of ligands on the surface of cell delivery systems influence their uptake efficiency and their mechanism of internalization [28]. The bimodal distribution of HSA-PEI-siRNA maybe a result of the combination of the large size of the ternary complex, heterogeneity in the MDA-MB-231 population, and effects associated with sedimentation of the polyplexes during transfection. For instance, caveolin1, part of caveolae-mediated endocytosis, is reported to be down-regulated during the activation of the cell cycle [29]. However, since HPMEC, a well-defined monodispersed cell population yielded a single transfected population, we suggest a possible role for differential expression of cell-surface proteins in different phases of the cell cycle in the internalization of the ternary complex rather than the size of the polyplex. Evidence in support of this conclusion can be found in a study by Ogris *et al*., who reported that the conjugation of PEI with tranferrin (Tf) could enhance gene delivery to tumors, despite the formation of complexes up to micrometer size [30]. In this study, the large HSA-bPEI-siRNA complexes also showed higher uptake. It is possible that a loose association of smaller polyplexes occur and this could be the reason for the higher efficiency. Further evidence in support of a clear role for HSA in transporting or facilitating siRNA internalization, was obtained by carrying out transfection in presence of serum. We hypothesized that since albumin is the dominant protein in serum, it would compete for trannporters on the cell surface thus dimninishing HSA-bPEI-siRNA ternary polyplex uptake and trasnfection. Interestingly, the transfection efficiency of the ternary complex was indeed diminished as postulated in presence of serum (S2 Fig).

### HSA increases gene silencing in MDA-MB-231 and HPMEC

**Fig 4:**
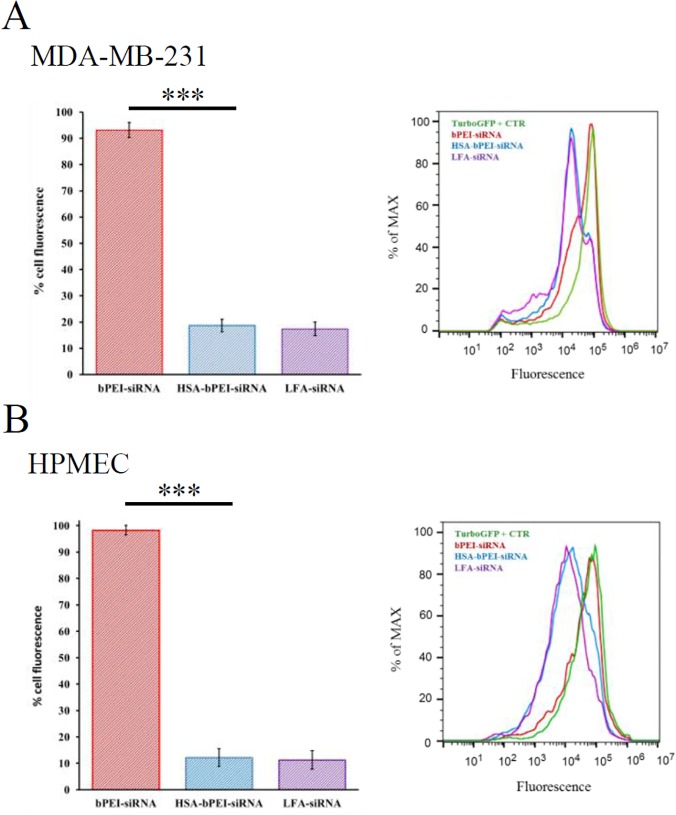
Protein knockdown efficiency in cells expressing TurboGFP. Cell fluorescence reduction was estimated to evaluate the percentage of TurboGFP silencing. Both cell lines showed improved siRNA efficiency compared to bPEI-siRNA control. A) MDA-MB-231 ***p<0.0005. B) HPMEC ***p<0.0005. The silencing efficiency resulted comparable with Lipofectamine2000-siRNA lipoplexes (LFA-siRNA). Results are shown as mean ± SD (n = 6).

To see if the increased uptake efficiency translates into higher availability of free siRNA, a gene silencing study was undertaken using cell lines expressing TurboGFP. In accordance with the higher internalization, HSA-bPEI-siRNA showed about 12 and 53 times more silencing than bPEI-siRNA, in MDA-MB-231 and HPMEC, respectively, and was comparable with Lipofectamine, as seen by the reduced TurboGFP fluorescence in cells transfected with the ternary complex. Similar results were also obtained in HPMEC, indicating that HSA complexation with bPEI-siRNA presents a general strategy for improving the efficiency in siRNA delivery. The lack of any appreciable gene silencing using bPEI-siRNA may be attributed to poor siRNA internalization. In MDA-MB-231, 62 ± 1.4% of the cell population was efficiently silenced with HSA-bPEI-siRNA complexes, with cell fluorescence reduction of 81 ± 2.4% (Fig 4A). For HPMEC, 69 ± 2.0% of cells showed efficient knockdown, having fluorescence reduction of 88 ± 3.3% for HSA-bPEI-siRNA (Fig 4B). Lipofectamine2000-siRNA showed similar results, in terms of effectively silenced population and knockdown efficency. The contribution of differences in cell viability, in the observed higher transfection efficiency with the ternary system, can be eliminated, as cells exposed to HSA-bPEI-siRNA and bPEI-siRNA complexes showed comparable viability in MTT assay (S3 Fig). The positive impact of the incorporation of HSA with PEI appear consistent with the previous studies involving DNA delivery in endothelial and epithelial cells [17–18] and with the study of Abbasi et al. where siRNA delivery in tumor cells was accomplished using PEI-coated HSA nanoparticles [19].

### Uptake Mechanism of HSA-bPEI-siRNA complexes

**Fig 5:**
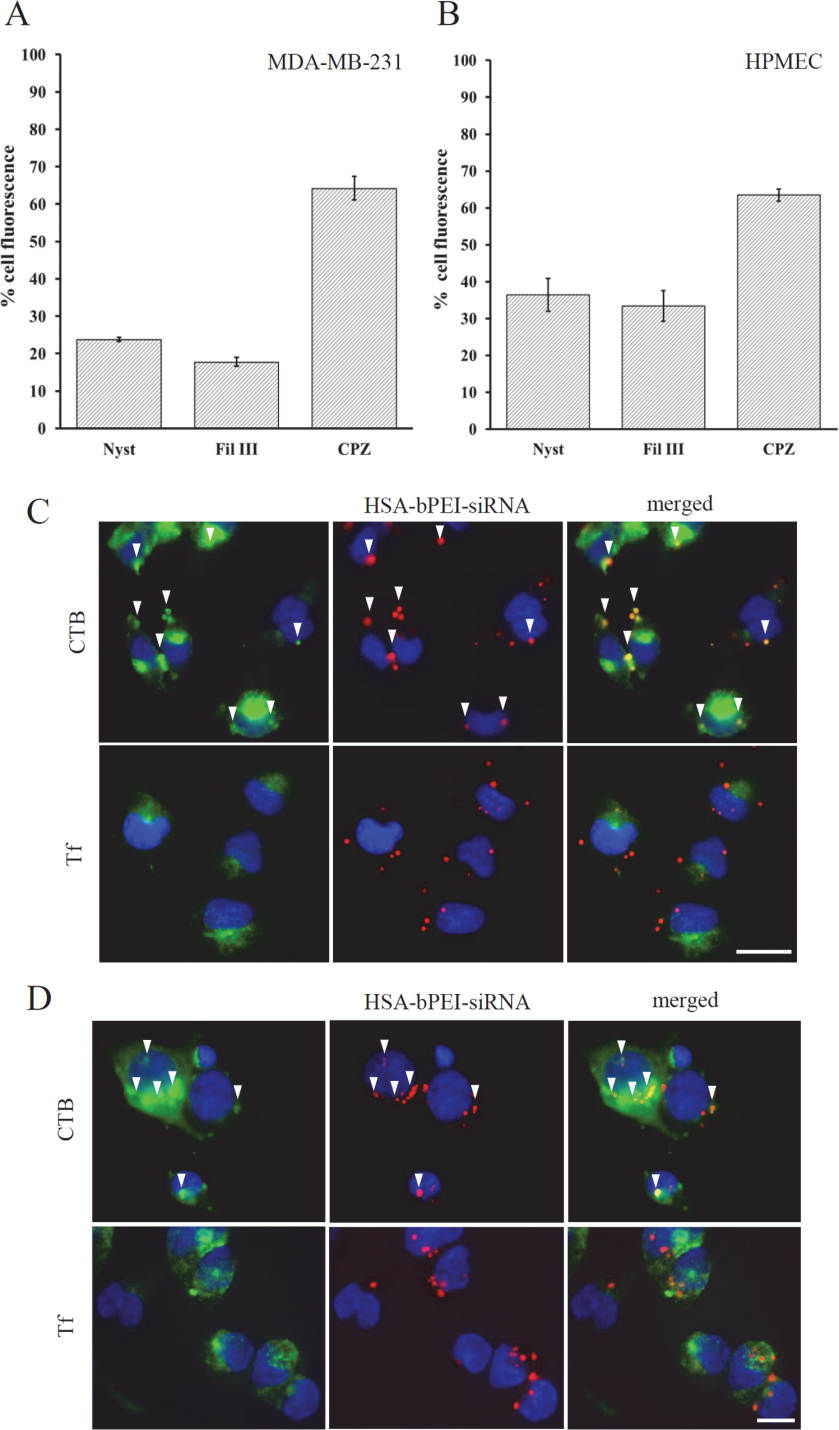
Uptake mechanism of HSA-bPEI-siRNA complexes in MDA-MB-231 and HPMEC. The uptake inhibition was quantified in presence of Nystatin (Nyst), Filipin III (Fil III) and Chlorpromazine (CPZ). Fluorescence reduction was evaluated in FACS. A) MDA-MB-231; B) HPMEC. Results are shown as mean ± SD (n = 3). From the study, partial involvement of clathrin-mediated pathway was observed, with around 30% inhibition by CPZ, and low colocalization with Tf. Predominant activation of caveolae-mediated endocytosis resulted in the internalization of HSA-bPEI-siRNA complexes, in both cell lines. Colocalization study in fluorescence microscopy with uptake markers cholera toxin b (CTB), marker of caveolae-mediated pathway, and transferrin (Tf), marker of clathrin-mediated pathway, is shown in C) MDA-MB-231 cells; D) Colocalization results in HPMEC. White arrows indicate points of colocalization. Scale bar: 20μm.

In order to understand which uptake mechanism mediates the higher siRNA delivery efficiency of HSA-bPEI-siRNA, the involvement of clathrin-mediated endocytosis and caveolae-mediated endocytosis was elucidated. The size of the complexes influences their entry in cells and their intracellular trafficking. Previous studies have demonstrated that nanospheres with diameter lower than 200 nm were preferentially internalized by chlatrin mediated pathway, while particles higher than 500 nm followed the activation of caveolae or “caveosomes”, derived from multiple caveolae assemblies on the cellular surface [31–32]. We investigated the uptake inhibition of siRNA-labeled complexes in presence of Chlorpromazine (CPZ), a known inhibitor of the clathrin pathway, and Nystatin (Nyst) and Filipin III (Fil III), both cholesterol depletors, that block the formation of caveolae.The inhibitors concentrations were chosen so as to have minimal impact on cell viability (S4 Fig). We observed that the ternary complexes are primarily taken up by the caveolae uptake mechanism in both cells, which this uptake pathway was more pronunced in MDA-MB-231. The uptake through clathrin mediated endocytosis was around 30% in MDA-MB-231 and HPMEC (Fig 5A and 5B). Colocalization with cholera toxin b (CTB) in fluorescence microscopy further confirmed the intracellular trafficking through caveolae (Fig 5C and 5D).

It is well known that endothelial cells have high expression of caveolae [33]. Also multidrug resistant (MDR) cancer cells have been shown to significantly overexpress caveolae [34]. Since we have shown that the particles appear to be taken up through caveolae mediated endocytosis, the higher efficiency could be due to the fact that this pathway is able to bypass lysosomal degradation [35–31]. With regards to albumin trafficiking into cells, Gp60, a protein that is associated with caveolae complexes has been shown to aid in the transport of albumin across endothelial and epithelial barriers [36–27]. Therefore, the specific role of gp60 in enhancing the uptake efficiency of the ternary complex needs to be investigated further.

**Fig 6:**
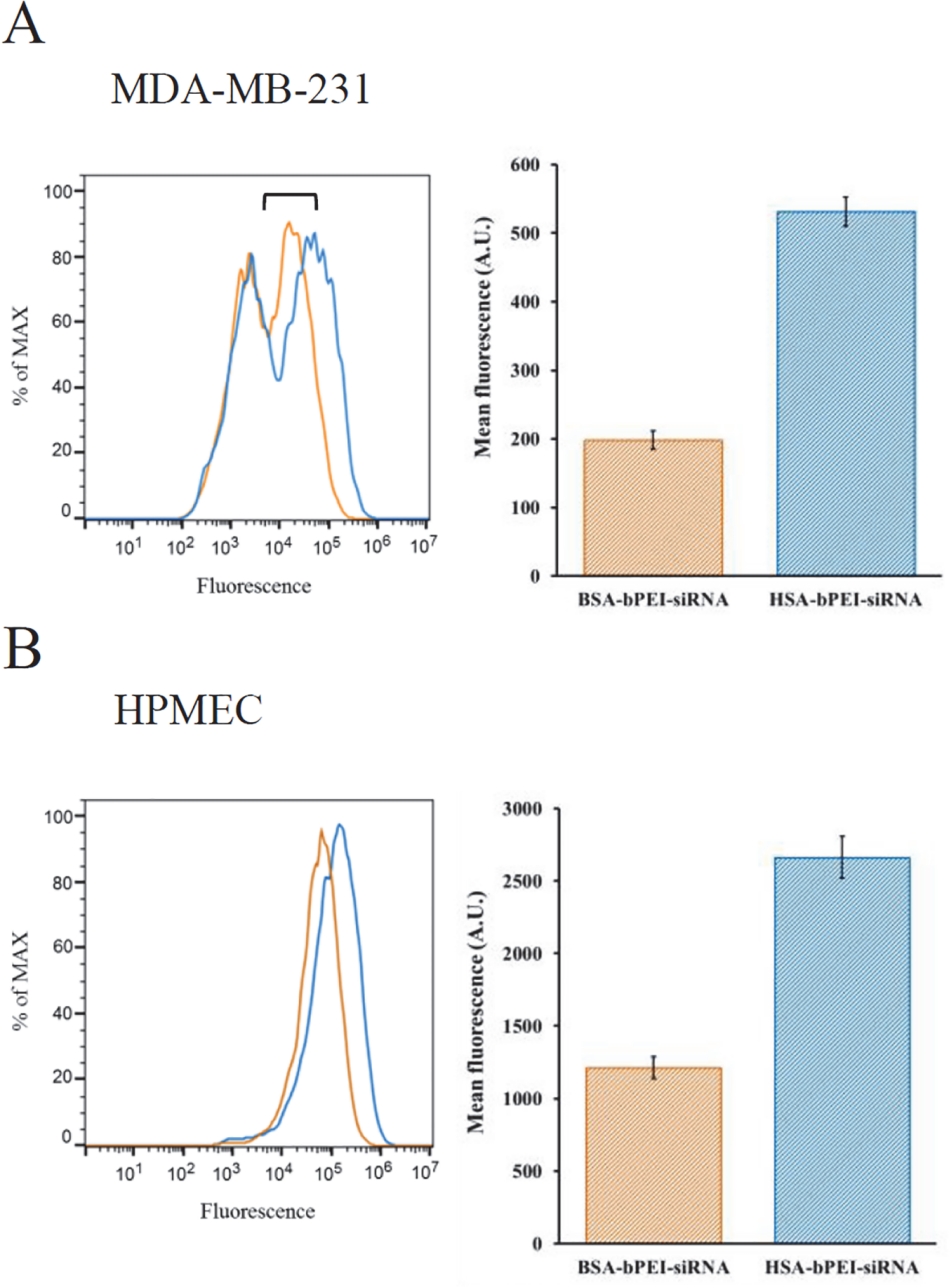
Uptake efficiency comparing complexes formed with BSA and HSA. Uptake efficiency was evaluated by FACS. (A) Study in MDA-MB-231, p<0.005; B) Study in HPMEC, p<0.01. Results are shown as mean ± SD (n = 3). Complexes formed with BSA showed reduced internalization compared with HSA, confirming the role of HSA in improving siRNA delivery in both cell lines.

To further elaborate the role of albumin in the uptake of HSA-bPEI-siRNA complexes, we substituted HSA with bovine serum albumin (BSA) and we made an interesting observation, that the uptake efficiency was diminished by over 2 fold in both cell lines. Although HSA and BSA yielded complexes comparable in size, they showed different uptake profiles (Fig 6) and this clearly shows that human albumin confers specificity in the uptake in human cells. Similarly, it has been postulated that the higher efficiency observed when paclitaxel is covalently linked to HSA (Abraxane) is due to the active uptake of albumin, through the gp60 receptor [26].

## Conclusions

In this study, we evaluated the role of native HSA in mediating the uptake and silencing of siRNA, towards cells expressing TurboGFP. It was observed that the presence of albumin on the exterior of the bPEI-siRNA polyplexes dramatically improve uptake and gene silencing in both human endothelial and tumor epithelial cells. Inhibition studies suggest a role for caveolae mediated endocytosis in the uptake of the ternary complexes. This system offers significant promise for the in vitro delivery of siRNA.

## Supporting Information

S1 Fig HSA-bPEI-siRNA stability. Ternary complexes stability was evaluated by size measurements by DLS. The study was performed at different time-points: 30 minutes, 1 hour, 2 hours and 4 hours. At the time of 6 hours the complexes were not more detectable, showing stability for up to 4 hours. Results are shown as mean ± SD (n = 3).
(TIF)

S2 Fig Cell uptake study comparing HSA-bPEI-siRNA and bPEI-siRNA tranfection in medium with and without fetal bovine serum (FBS). The uptake of the ternary complexes resulted significantly inhibited by serum in A) MDA-MB-231 ***p<0.0005; B) HPMEC **p<0.005. Results are shown as mean ± SD (n = 3). As control, bPEI-siRNA uptake efficiency in presence of FBS was also tested, without observing a statistically significant reduction.
(TIF)

S3 Fig MTT test showing cell viability after transfection. Study performed at the time-point of 72 hours, after transfection with HSA-bPEI-siRNA, bPEI-siRNA and LFA-siRNA complexes in A) MDA-MB-231; B) HPMEC. The percentage of cell viability was normalized with untreated cells. Results are shown as mean ± SD (n = 3). No contribution in cell viability was given to bPEI-siRNA by the ternary complex, but increased cell viability was observed in comparison with LFA-siRNA lipoplexes (***p<0.0005 in MDA-MB-231 and **p<0.005 in HPMEC), considering the similar protein silencing efficiency.
(TIF)

S4 Fig MTT test showing cell viability after incubation with inhibitors, at the concentration used. Cell viability in A) MDA-MB-231; B) HPMEC. Results are shown as mean ± SD (n = 3). Cell viability resulted in both cell lines between 80 and 90% in comparison with untreated cells.
(TIF)
