# Supplementary figures and images for: Enhanced Gene Silencing through Human Serum Albumin-Mediated Delivery of Polyethylenimine-siRNA Polyplexes
Source: PLoS One. 2015 Apr 9;10(4):e0122581. doi: 10.1371/journal.pone.0122581 (PMC4391875; doi:10.1371/journal.pone.0122581)

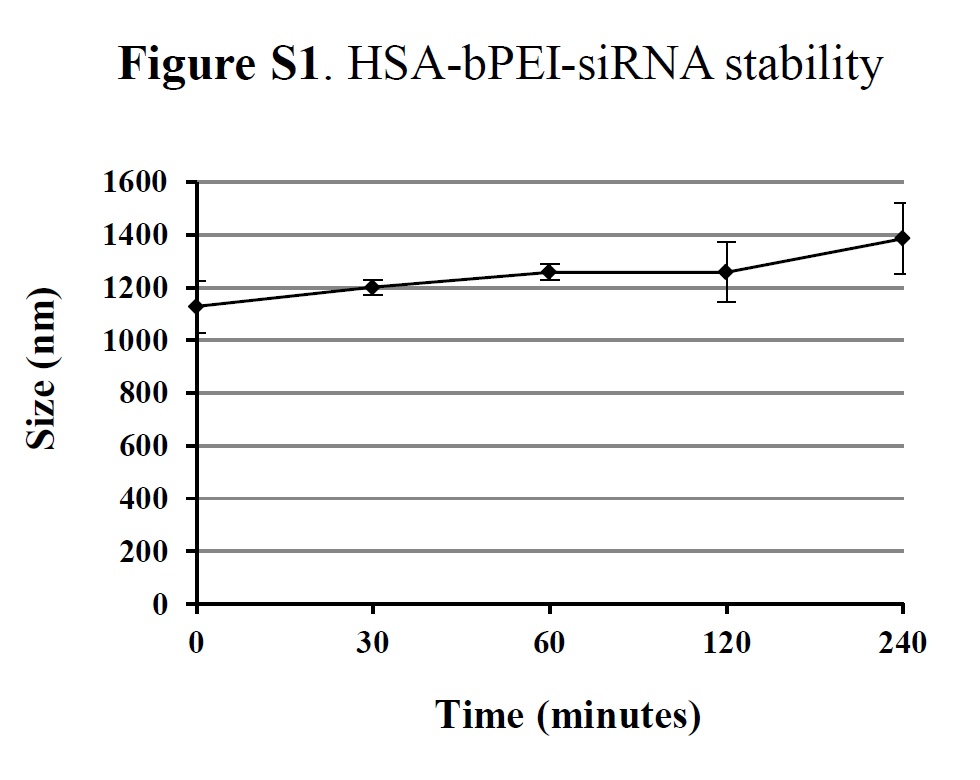

Supplement: S1 Fig — Ternary complexes stability was evaluated by size measurements by DLS. The study was performed at different time-points: 30 minutes, 1 hour, 2 hours and 4 hours. At the time of 6 hours the complexes were not more detectable, showing stability for up to 4 hours. Results are shown as mean ± SD (n = 3). (TIF) [file pone.0122581.s001.tif]

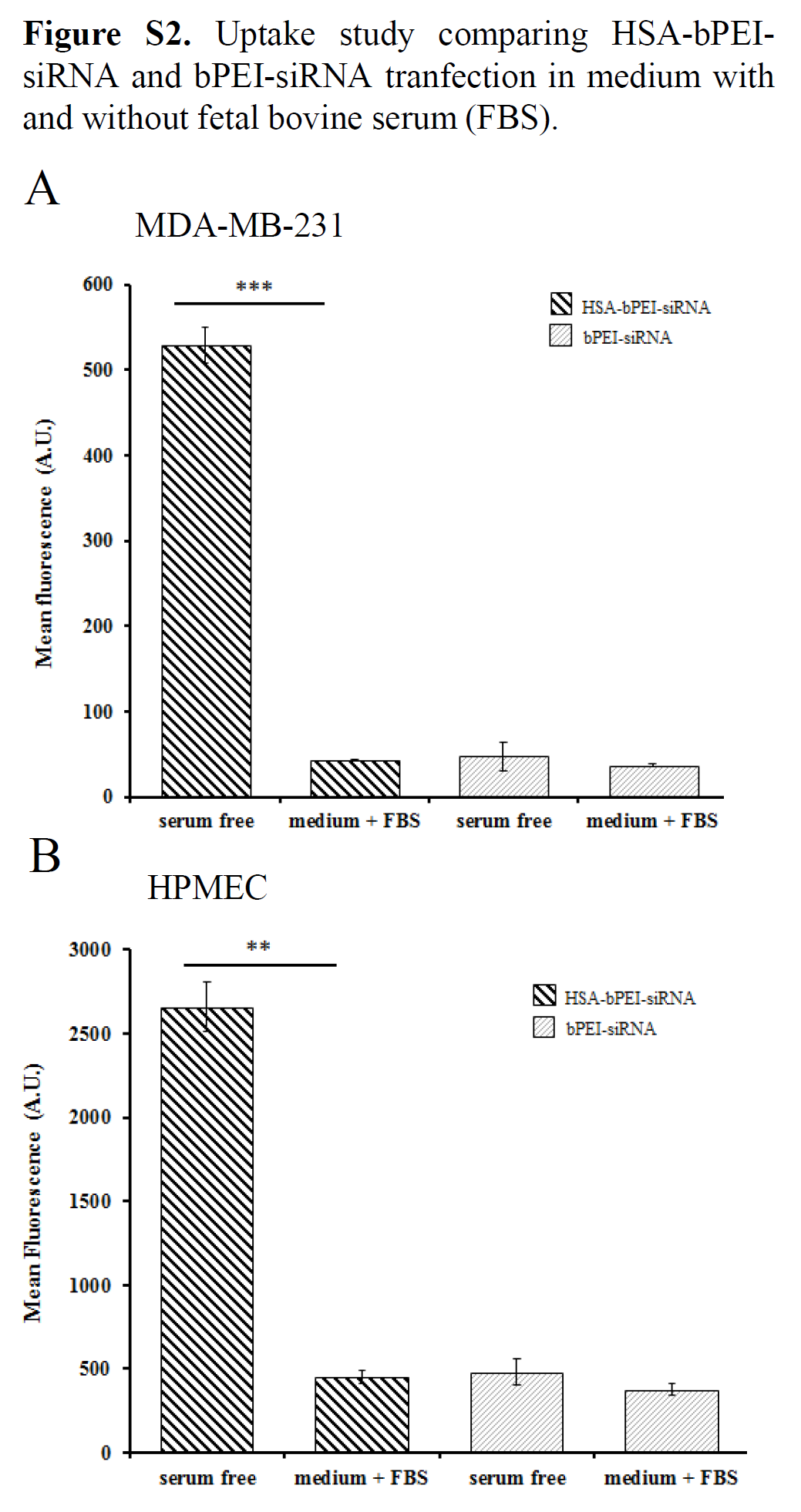

Supplement: S2 Fig — The uptake of the ternary complexes resulted significantly inhibited by serum in A) MDA-MB-231 ***p<0.0005; B) HPMEC **p<0.005. Results are shown as mean ± SD (n = 3). As control, bPEI-siRNA uptake efficiency in presence of FBS was also tested, without observing a statistically significant reduction. (TIF) [file pone.0122581.s002.tif]

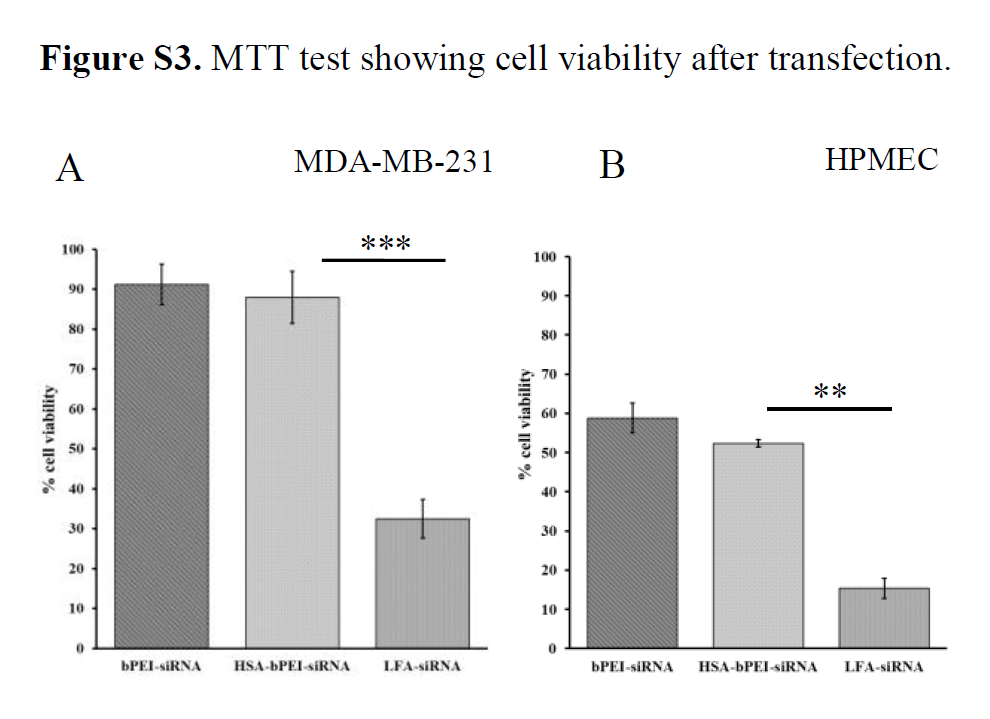

Supplement: S3 Fig — Study performed at the time-point of 72 hours, after transfection with HSA-bPEI-siRNA, bPEI-siRNA and LFA-siRNA complexes in A) MDA-MB-231; B) HPMEC. The percentage of cell viability was normalized with untreated cells. Results are shown as mean ± SD (n = 3). No contribution in cell viability was given to bPEI-siRNA by the ternary complex, but increased cell viability was observed in comparison with LFA-siRNA lipoplexes (***p<0.0005 in MDA-MB-231 and **p<0.005 in HPMEC), considering the similar protein silencing efficiency. (TIF) [file pone.0122581.s003.tif]

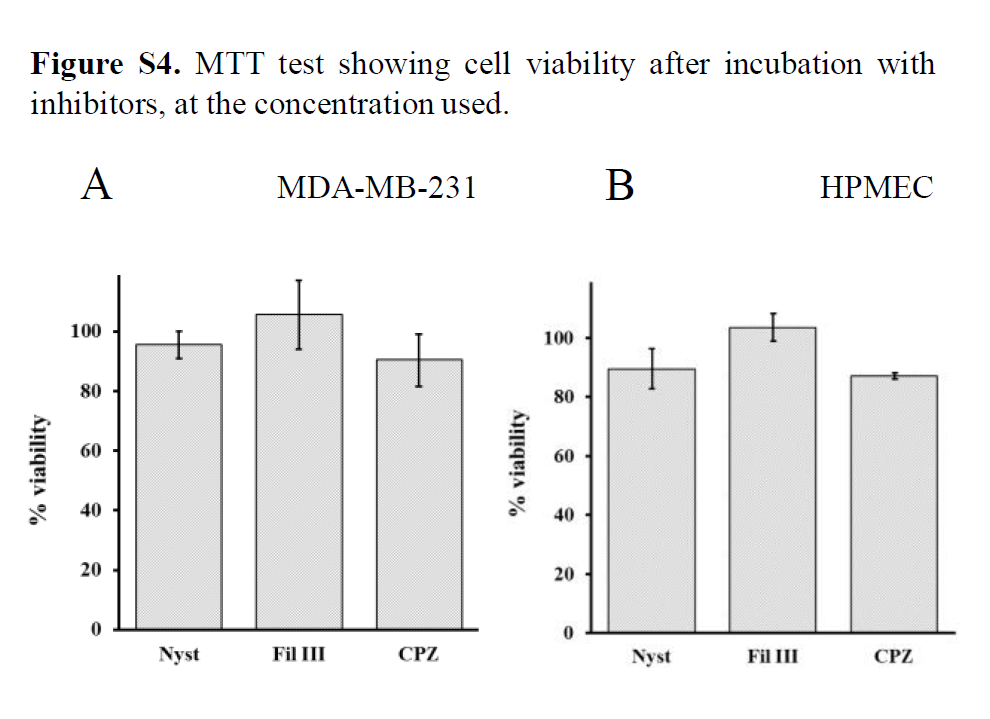

Supplement: S4 Fig — Cell viability in A) MDA-MB-231; B) HPMEC. Results are shown as mean ± SD (n = 3). Cell viability resulted in both cell lines between 80 and 90% in comparison with untreated cells. (TIF) [file pone.0122581.s004.tif]
